# Supplementary material for: M1C IS NECESSARY FOR DARAXONRASIB RESISTANCE OF NSCLC KRAS(G12C) MUTANT CELLS
Source: bioRxiv. 2026 Jun 23:2026.06.20.733526. Preprint. [Version 1] doi: 10.64898/2026.06.20.733526 (PMC13334778; doi:10.64898/2026.06.20.733526)
Supplement: Supplement 1 — Supplemental Figure S1. Targeting RAS(ON) with TCIs induces M1C expression. A. H358 cells treated with 100 nM daraxonrasib for the indicated days were analyzed for M1C transcripts. The results (mean±SD of 4 determinations) are expressed as relative levels compared to that obtained for control cells (assigned a value of 1). B. H2122 cells treated with the indicated concentrations of daraxonrasib for 48 hours were analyzed for M1C transcripts. The results (mean±SD of 4 determinations) are expressed as relative levels compared to that obtained for control cells (assigned a value of 1). C. Lysates from MGH1112 cells treated with 1 μM daraxonrasib for 48 hours were immunoblotted with antibodies against the indicated proteins. D and E. H358 (D) and H2122 (E) cells treated with the indicated concentrations of RMC-7977 for 48 hours were analyzed for M1C transcripts. The results (mean±SD of 4 determinations) are expressed as relative levels compared to that obtained for control cells (assigned a value of 1). F and G. H358 cells treated with the indicated concentrations of RMC-7977 and GO-203 (F) or RMC-7977 and C-11 (G) for 48 hours were analyzed for viability by Alamar blue staining. Indicated are the combination indices determined using Bliss scores. H. Lysates from H358-SR cells treated with 1 μM daraxonrasib for 48 hours were immunoblotted with antibodies against the indicated proteins. I. H358-SR cells treated with the indicated concentrations of daraxonrasib and C-11 for 48 hours were analyzed for viability by Alamar blue staining. Indicated are the combination indices determined using Bliss scores. Supplemental Figure S2. M1C confers resistance to RAS(ON) TCIs. A. H358 and H358/DAR-R cells treated with the indicated concentrations of daraxonrasib for 48 hours were analyzed for viability by Alamar blue staining. Indicated are the IC50 values. B. H358/DAR-R/tet-CshRNA and H358/DAR-R/tet-MUC1shRNA cells treated with vehicle or DOX for 7 days were analyzed for M1C transcr [file media-1.pdf]

Supplementary Figure 1

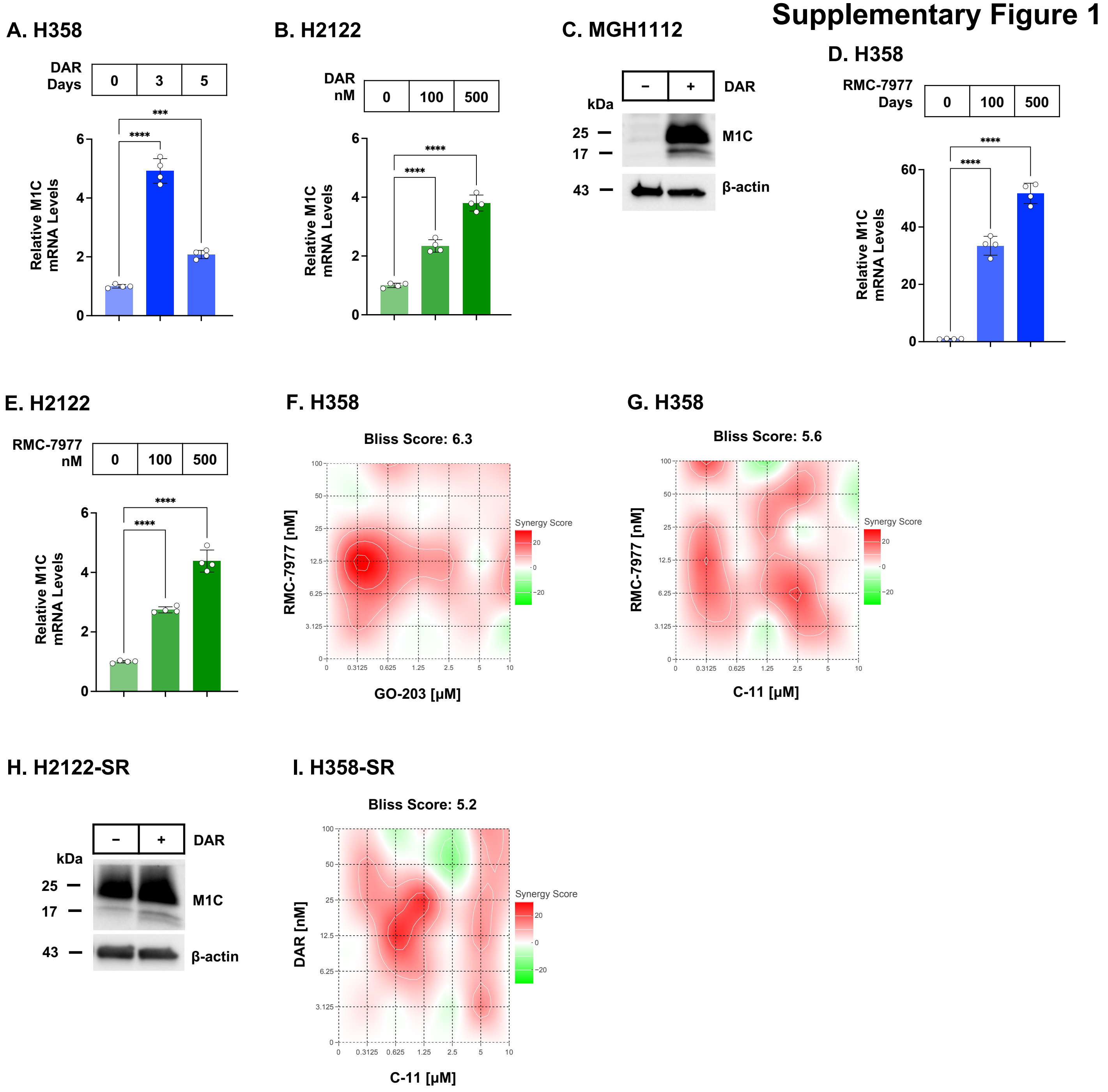

Supplementary Figure 2

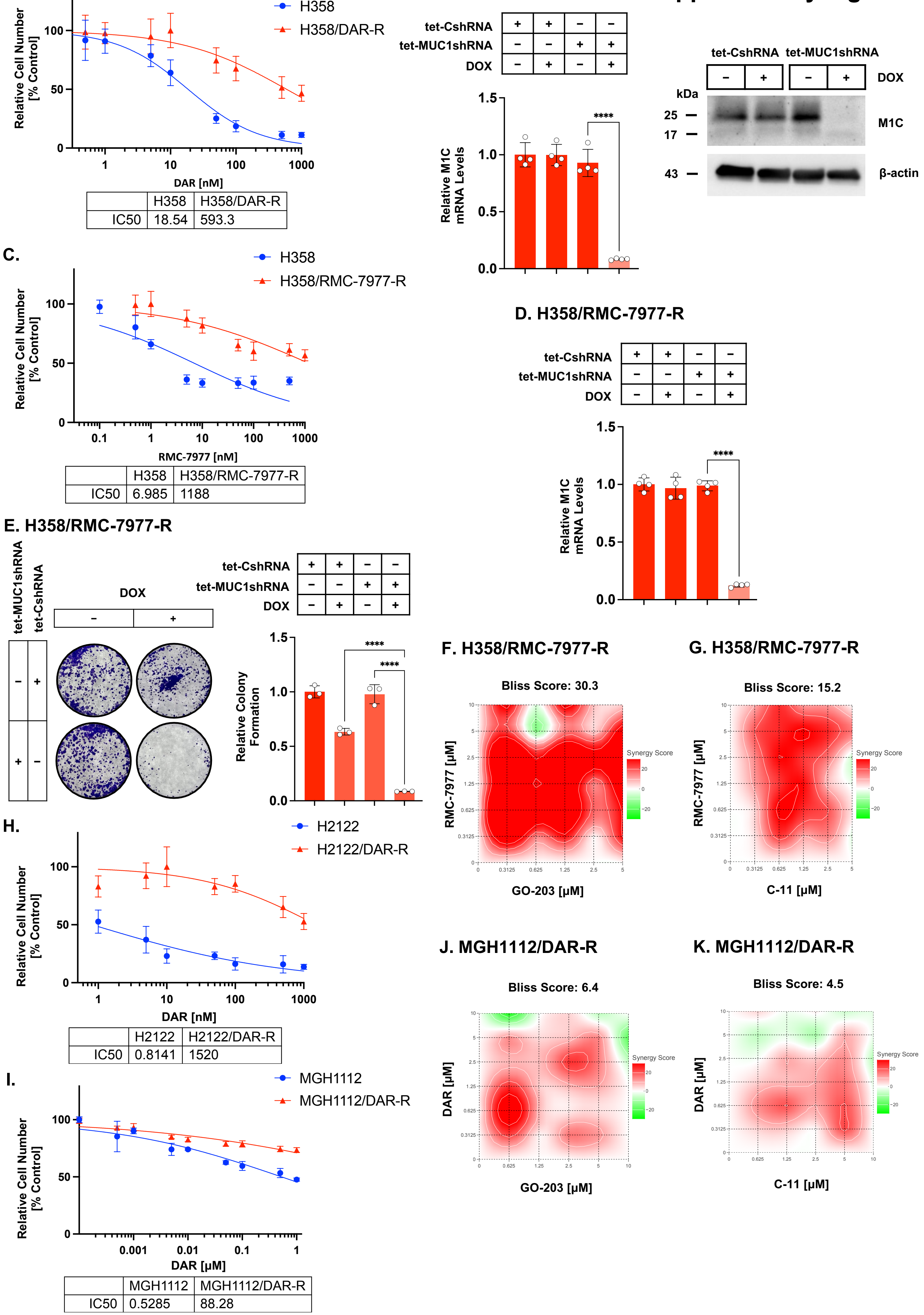

Supplementary Figure 3

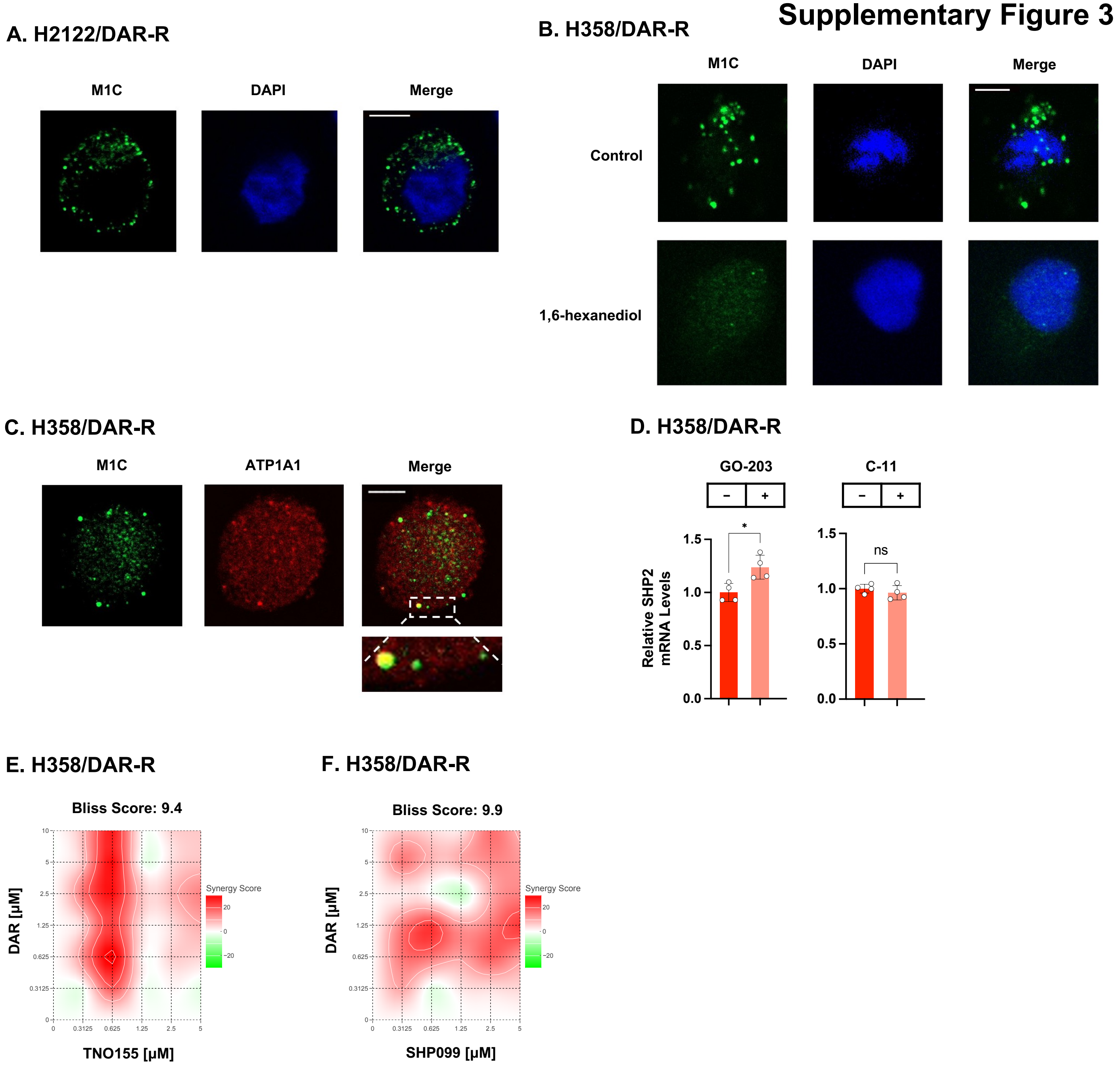

Supplementary Figure 4

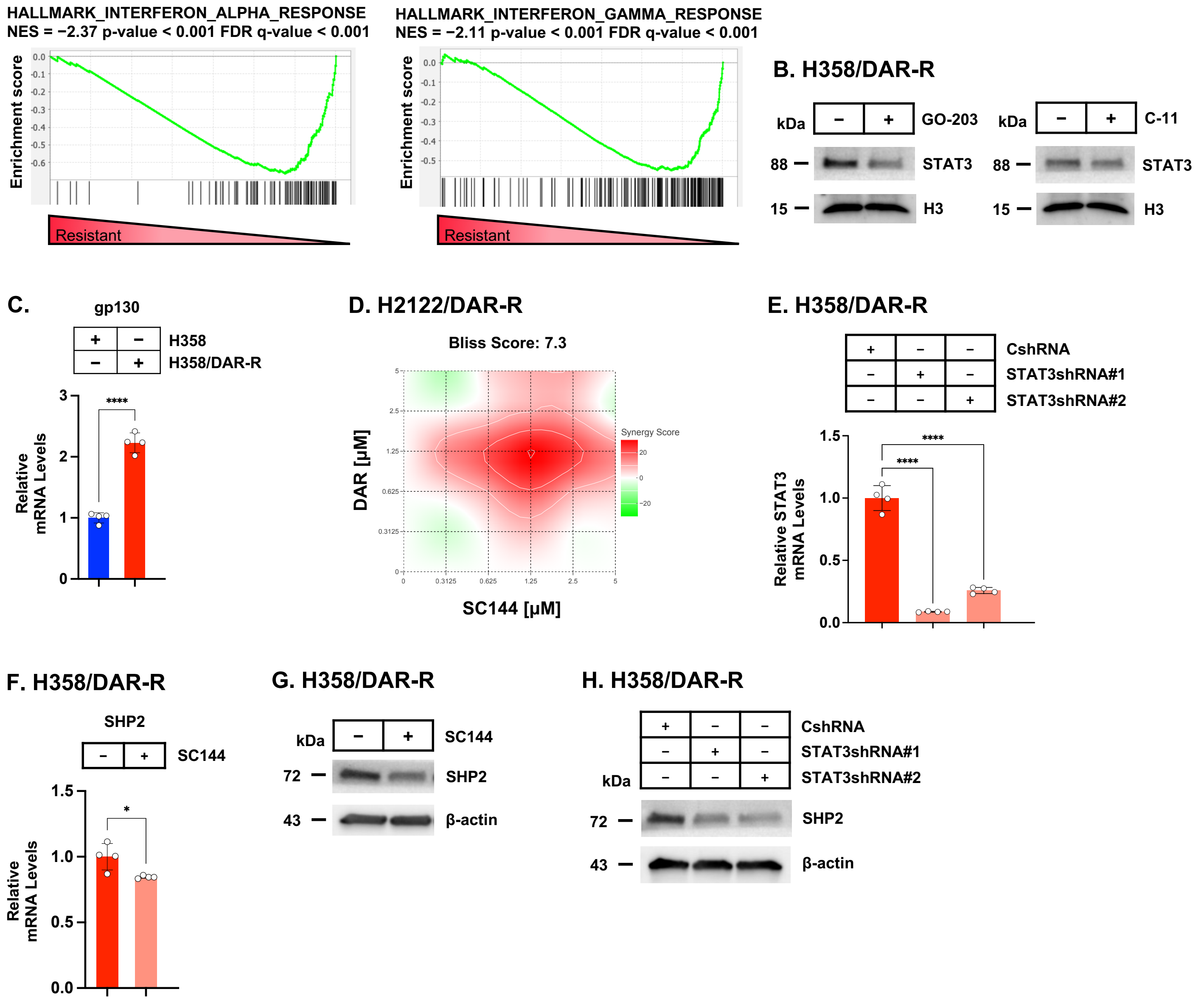

Supplementary Figure 5

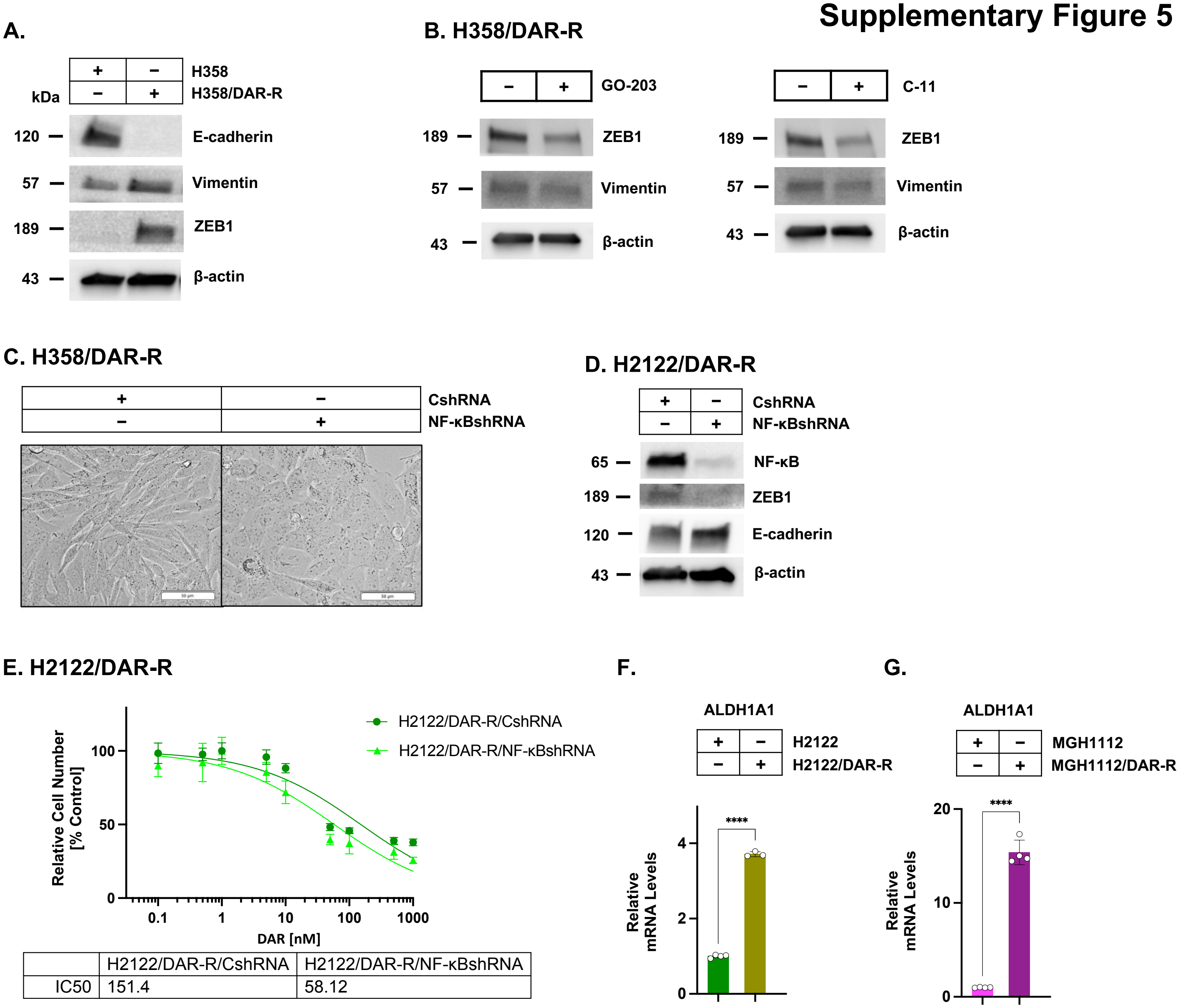

	H2122/DAR-R/CshRNA	H2122/DAR-R/NF-κBshRNA
IC50	151.4	58.12

Supplementary Figure 6

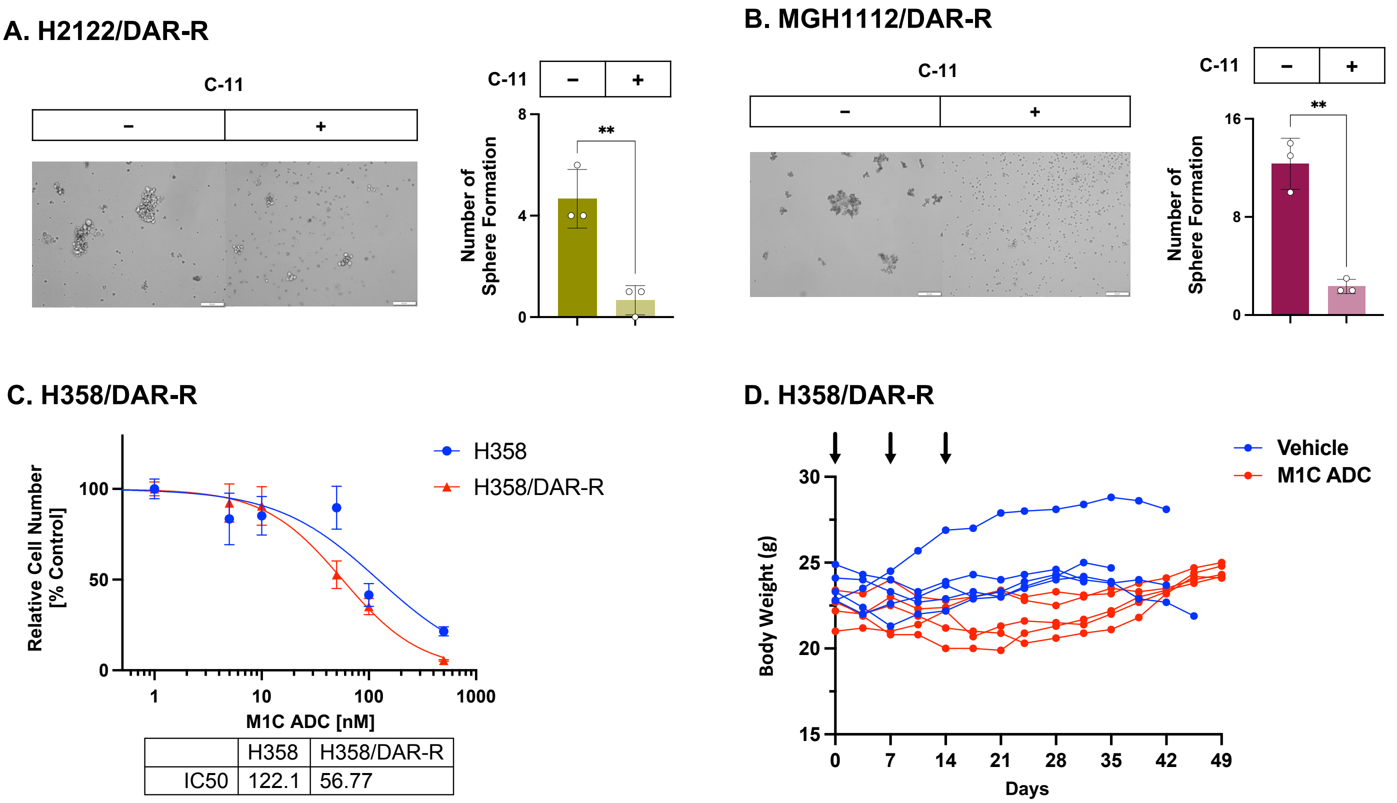

	H358	H358/DAR-R
IC50	122.1	56.77

Supplementary Figure 7

A. H358/DAR-R vs. H358-SR

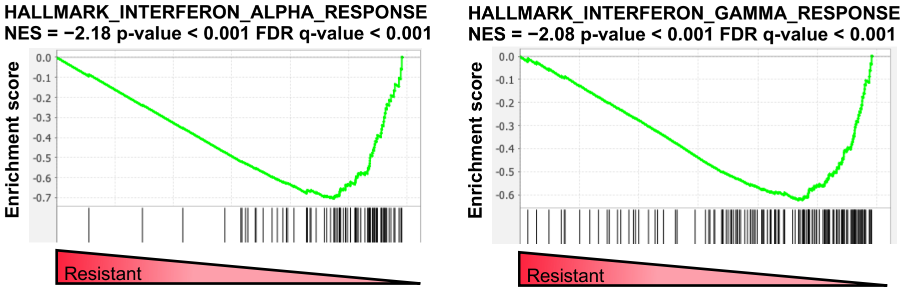

B. H358/DAR-R vs. H358-SR

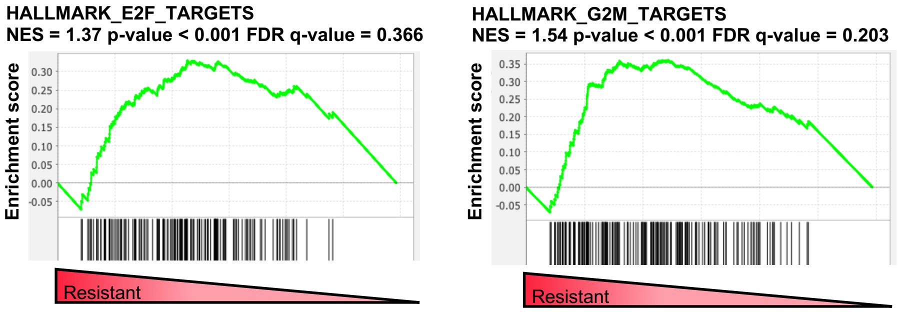

C.

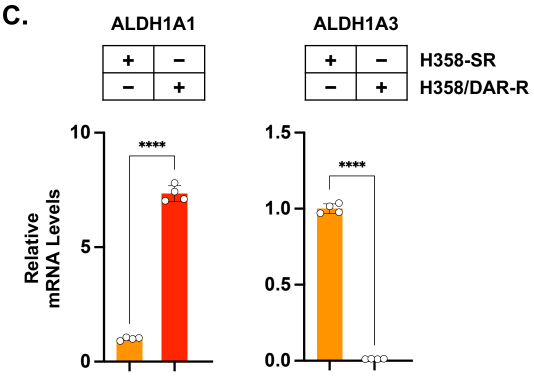

D.

RESISTANCE	SOTORASIB	DAR
**Transcriptome**		
IFN	+	−
G2M	−	+

**Upregulation**		
OSM	−	+
gp130	−	+
STAT1	+	−
STAT3	−	+
EMT	+	+
