# Supplementary material for: M1C IS NECESSARY FOR DARAXONRASIB RESISTANCE OF NSCLC KRAS(G12C) MUTANT CELLS
Source: bioRxiv. 2026 Jun 23:2026.06.20.733526. Preprint. [Version 1] doi: 10.64898/2026.06.20.733526 (PMC13334778; doi:10.64898/2026.06.20.733526)
Supplement: 1 [file NIHPP2026.06.20.733526V1-supplement-1.pdf]

bioRxiv preprint doi: https://doi.org/10.64898/2026.06.20.733526; this version posted June 23, 2026. The copyright holder for this preprint (which was not certified by peer review) is the author/funder, who has granted bioRxiv a license to display the preprint in perpetuity. It is made available under a CC-BY 4.0 International license.

Supplemental Table S1. Primers used for qRT-PCR analysis.

**M1C**	**FWD**	TACCGATCGTAGCCCCTATG
	**REV**	CTCACCAGCCCAAACAGG
**SHP2**	**FWD**	GACTTTTGGCGGATGGTGTTCC
	**REV**	CGGCGCTTTCTTTGACGTTCCT
**IL-6**	**FWD**	AGAGGCACTGGCAGAAAACAAC
	**REV**	AGGCAAGTCTCCTCATTGAATCC
**OSM**	**FWD**	GTGAACGGAACAGGTCTCCC
	**REV**	CAAGGACCAGACCTTGTCAG
**LIF**	**FWD**	GGCCCGGACACCCATAGACG
	**REV**	CCACGCGCCATCCAGGTAAA
**gp130**	**FWD**	CCATAGTCGTGCCTGTTTGC
	**REV**	CTTGGAGGAGTGTGAGGTGAC
**STAT3**	**FWD**	GGGAAGAATCACGCCTTCTAC
	**REV**	ATCTGCTGCTTCTCCGTCAC
**ALDH1A1**	**FWD**	CCTGTCCTACTCACCGATTTG
	**REV**	TCCTCCTTATCTCCTTCTTCTACC
**ALDH1A3**	**FWD**	TGGATCAACTGCTACAACGC
	**REV**	CACTTCTGTGTATTCGGCCA
**β-actin**	**FWD**	GATGAGATTGGCATGGCTTT
	**REV**	CACCTTCACCGTTCCAGTTT
